# Change of Anesthesia Management for a Patient Undergoing CABG by an Incidental Finding of a Genetic Variant Associated with Malignant Hyperthermia

**DOI:** 10.1155/2019/3189719

**Published:** 2019-03-19

**Authors:** Trey B. Creech, Li Zhang

**Affiliations:** Anesthesiology Department, Geisinger Medical Center, Danville, PA, USA

## Abstract

Malignant hyperthermia (MH) is a rare life-threatening hypermetabolic muscular disorder with a high mortality rate. Three genes,* RYR1*,* CACNA1S*, and* STAC3*, have been associated with MH susceptibility. Multiple genetic variants have been identified in these three genes. Some of those variants were pathogenic, but many others are yet to be tested. Such uncertainty can make it challenging for anesthesia providers as there is currently no anesthesia guideline for each genetic variant in patients who have neither clinical nor family history of MH. With the increasing popularity of whole exome sequencing, anesthesia providers will likely face such challenges more often as many patients may have genetic variations of unknown clinical significance in their* RYR1*,* CACNA1S*, or* STAC3* genes. Here we describe change of anesthesia management for a patient who had an incidental finding of a genetic variant in* RYR1* gene undergoing an elective coronary artery bypass surgery.

## 1. Introduction

Malignant hyperthermia (MH) is a life-threatening hypermetabolic muscular disorder with a mortality rate as high as 9.5% [1]. It is thankfully a rare disorder with events approximately 1:40,000 in adults and 1:15,000 in pediatrics [2]. It is traditionally thought of as autosomal dominant but exhibits incomplete penetrance [3]. Three genes,* RYR1* (ryanodine receptor 1),* CACNA1S* (calcium voltage-gated channel subunit *α*1 subunit S receptor), and* STAC3* (SH3 and cysteine rich domain 3 protein), have been associated with MH susceptibility [4]. Multiple genetic variants have been identified in these three genes. Some of those variants were proved to be pathogenic, but many others are yet to be tested. Such uncertainty can make it challenging for anesthesia providers as there is currently no anesthesia guideline for each genetic variant in patients who have neither clinical nor family history of MH. Here we describe the management of a patient who had an incidental finding of a genetic variant in* RYR1* gene but without clinical or family history of MH undergoing an elective CABG (coronary artery bypass grafting) with CBP (cardiopulmonary bypass).

## 2. Case Report

A 70-year-old male with history of angina, 3-vessel CAD (coronary artery disease), TIA (transient ischemic attack), hyperlipidemia, and gastric bypass presented for an elective CABG. The patient had no family history of MH. He had no problems with general anesthesia with sevoflurane or desflurane previously. Prior to this surgery, he was enrolled in Geisinger MyCode program, which is part of a public health and research initiative at our institution that offers whole exome genetic screening for all participants [5, 6]. It was through this program that a genetic variant in* RYR1* gene (c.1840 C>T p.Arg614Cys) was incidentally identified. There are currently more than 400 genetic variants identified in* RYR1* with 48 known to cause MH according the European Malignant Hyperthermia Group (https://www.emhg.org; accessed December 26, 2018). The variant in this patient happened to be one of those 48 pathogenic mutations. We decided to proceed as a MH positive case although this patient had neither clinical nor family history of MH. The anesthesia workstation was prepared with a new circuit, CO_2_ absorbent, charcoal filters, and removal of the vaporizers. The perfusionist was reminded that this patient should be treated as MH positive and no volatile anesthetics should be added to the circuit when CPB was needed. The patient was induced with nondepolarizing NMB rocuronium. A balanced anesthetic was maintained with fentanyl, midazolam, and infusion of propofol. A BIS (bispectral index) monitor was applied throughout the surgery to monitor the depth of anesthesia. The case was originally scheduled for off-pump but the LIMA (left internal mammary artery) graft was torn during repositioning of the heart and urgent CPB was initiated. Anesthesia was maintained with midazolam and fentanyl during CBP. The patient had moderate microvascular coagulopathy after CPB with a normal ACT (activated clotting time) which was treated with platelets and cryoprecipitate. The patient's MH susceptibility and its clinical relevance were discussed with the ICU (intensive care unit) team during postoperative handoff. Additionally, this patient's MH susceptibility was discussed with the on-call anesthesiologist in case an emergent operation was necessary overnight. The patient was extubated postoperatively with an uncomplicated postoperative course. He had no recall of the surgery.

## 3. Discussion

*RYR1* mutations are the most common genetic variants for the MH phenotype. There are currently more than 400 genetic variants identified in* RYR1*. The clinical significance of many of these mutations is not well understood. With the rise in whole exome sequence, many patients being screened primarily for non-MH related conditions may be marked as MH susceptible due to the prevalence of* RYR1* and* CACNA1S* mutations with undetermined clinical significance [7]. The risk of using a triggering agent in a patient with a known pathogenic variant is not justifiable. However, it becomes more complicated when considering mutations of unknown clinical significance. The benefits of pursuing nontriggering anesthetics in these situations are reasonable but not without consequences. Treating all as MH susceptible may preclude patients or their families from certain occupations, insurance policies, or even concerns for overseas travel [7]. Other disadvantages could include expensive anesthetics with more laborious room turnovers as well as inability to undergo procedures at surgical centers. In our case, the patient has a* RYR1* mutation that is known to be pathogenic and thus the decision to treat him as MH susceptible was not difficult. Unfortunately, there is no clear anesthesia guideline to date for many other mutations of unknown clinical significance. Establishment of a guideline will be helpful for anesthesia providers to manage patients with those genetic variants. As for now, we encourage that the anesthesiologists weigh the risks and benefits of proceeding with triggering agents in these cases.

At our institution, upon identification of any actionable mutations, the patients are notified and meet with genetic counselors. In the case of MH, they recommend to patients that they ensure clear communication of their susceptibility to their surgeon and anesthesia teams, notify family members of the result, and encourage genetic testing since first degree relatives are at a 50% risk of carrying a mutation, visit the Malignant Hyperthermia Association for the US website to educate themselves, and consider wearing a medical alert bracelet.

Muscle contracture testing may help confirm suspected pathogenic genes associated with MH. However, the muscle contracture testing has a sensitivity of 92-97% with specificity of only 53-78% for the North American Malignant Hyperthermia Group [8]. At our institution, we do not routinely refer patients with genetic variants associated with MH for muscle contracture testing.

CPB can make the identification of unanticipated MH more difficult due to various physiologic perturbations associated with initiation and cessation of CPB. The most common and reliable sign of MH while on CPB is an unexplained elevated PaCO_2_ or an unexplained, increasing trend of metabolic acidosis. Other signs may include a low mixed SvO_2_, which is more readily accessible in those lined for cardiac cases, as well as typical signs such as hyperkalemia, myoglobinuria, and muscle rigidity. Hyperthermia may not be as prevalent due to cooling associated with CPB but unexplained hyperthermia after cessation of CPB should raise clinical suspicion [9]. Most patients undergoing CPB for CABG have significant comorbidities with less physiologic reserve making their clinical course even more tenuous if afflicted with a MH crisis. Metabolic acidosis from MH will lead to a decreased responsiveness to vasopressors in the hemodynamically unstable patient which often occurs transiently after cessation of CPB. An elevated CO_2_ will lead to increased pulmonary hypertension and right ventricular strain which can be problematic in those with preexisting dysfunction. If an MH event occurs peri-CPB, it is often impossible or impractical to abort the surgical procedure at that time. Additionally, there are no clear recommendations regarding the pharmacokinetics of dantrolene while on CPB [10].

A case series by Metterlein et al. described the measures to prevent an episode of MH in 14 patients known or suspected to be MH susceptible undergoing procedures requiring CPB. All of these cases avoided triggering agents, used decontaminated anesthesia machines and oxygenators, and maintained anesthesia with opiates and intravenous hypnotics. Five of these 14 cases were given prophylactic dantrolene which is not currently recommended. Additionally, the authors suggested to slowly and carefully rewarm patients to 36°C as rewarming may precipitate a crisis in the absence of triggering agents [9].

The avoidance of volatile anesthetics, as required in patients susceptible to MH, is unlikely to affect the outcomes for CABG. There was thought to be a cardioprotective property of volatile anesthetics with patients undergoing CABG. However, a more recent study by Flier et al. found that there were no differences in isoflurane-opioid versus Propofol-opioid for CABG cases in respect to postoperative troponins or short- and long-term morbidity and mortality [11].

## 4. Conclusion

In summary, MH is a severe hypermetabolic disorder associated with dysregulation of calcium release and triggering agents. CPB required in many cardiac surgeries presents additional challenges to recognize and treat MH. Prior uncomplicated anesthesia history and no family history of MH are unreliable predictors of a lack of MH susceptibility. With the increasing prevalence of routine genetic screening, more patients will be identified to carry genetic variants associated with MH. Some of these variants are known pathogenic and many others are with uncertain clinical significance. Establishment of an anesthesia guideline for these genetic variants will be helpful for anesthesia providers to manage patients who are identified preoperatively as a carrier. Vigilance for signs and symptoms and early discontinuation of triggering agents with administration of dantrolene remain the mainstays of MH management.
