# Corrigendum to “Change of Anesthesia Management for a Patient Undergoing CABG by an Incidental Finding of a Genetic Variant Associated with Malignant Hyperthermia”

**DOI:** 10.1155/2019/3605064

**Published:** 2019-11-20

**Authors:** Trey B. Creech, Li Zhang

**Affiliations:** Anesthesiology Department, Geisinger Medical Center, Danville, PA, USA

In the article titled “Change of Anesthesia Management for a Patient Undergoing CABG by an Incidental Finding of a Genetic Variant Associated with Malignant Hyperthermia” [1], the sentence “At our institution, we do not routinely refer patients with genetic variants associated with MH for muscle contracture testing,” should be removed from the Discussion, where the third paragraph should read as follows:

“Muscle contracture testing may help confirm suspected pathogenic genes associated with MH. However, the muscle contracture testing has a sensitivity of 92–97% with specificity of only 53–78% for the North American Malignant Hyperthermia Group [8]”.
